# Focal pulsed field ablation at the coronary sinus ostium in the presence of a left ventricular lead: A case report

**DOI:** 10.1016/j.hrcr.2026.06.001

**Published:** 2026-06-04

**Authors:** Benjamin Vögeli, Nicolas Schaerli, Patrick Badertscher, Michael Kühne, Christian Sticherling, Philipp Krisai

**Affiliations:** 1 Cardiology Division, Department of Medicine; 2 Cardiovascular Research Institute Basel, University Hospital Basel, University Basel, Basel, Switzerland

**Keywords:** Pulsed field ablation, Dual-energy catheter, Cardiac implantable device, Cardiac resynchronization therapy, Atrial fibrillation

**Key Teaching Points**

• Focal pulsed field ablation (PFA) using a dual-energy catheter can be safely performed at the coronary sinus ostium in close proximity to the left ventricular lead of a chronic resynchronization therapy system.
• The interaction between PFA systems and cardiac implantable electronic devices varies significantly across different platforms.
• Pre- and postprocedural device interrogation is mandatory to detect both immediate and delayed complications including changes in battery status, sensing parameters, and pacing thresholds.

## Introduction

Atrial tachycardias (ATs) are common in patients with structural heart disease and after cardiac surgery.^1^^,^^2^ Catheter ablation remains a cornerstone of treatment, but certain anatomic regions, such as the coronary sinus (CS) ostium, pose significant challenges owing to proximity to coronary arteries, atrioventricular node, and potentially cardiac implantable electronic devices (CIEDs), if in place.^3^^,^^4^

Pulsed field ablation (PFA), a nonthermal ablation modality based on electroporation, offers the potential for safe and precise tissue-specific lesion formation.^5^ However, data on its application in the CS ostium area with the presence of in situ chronic resynchronization therapy (CRT) leads remain limited. Case reports and small-case series have reported the potential of malfunction or permanent damage to CIEDs with PFA.^6^, ^7^, ^8^

Here we present the case of a patient with recurrent AT after redo aortic valve replacement for infectious endocarditis, managed successfully with PFA at the CS ostium in the presence of a CRT system.

## Case description

A 46-year-old man with a history of severe aortic stenosis underwent mechanical aortic valve replacement in July 2024. He subsequently developed recurrent cardioembolic events including ischemic strokes (April and May 2025) and non-ST elevation myocardial infarction attributed to embolism (June 2025). Clinical and imaging findings revealed prosthetic valve endocarditis, and the patient underwent urgent redo surgery with replacement of the mechanical valve by a biological prosthesis. Postoperatively, the patient developed sustained complete heart block requiring pacing resulting in implantation of a left bundle branch–optimized CRT pacemaker system (Generator U228 Visionist CRT-P, Boston Scientific Inc, Marlborough, MA) with right atrial (Medtronic 5076-52 CapSureFix Novus magnetic resonance imaging), left bundle branch area (Medtronic 383069 LEAD 383069 SELECT SEC), and quadripolar/left ventricular (LV) leads (Boston Scientific 1458Q QUARTET). The postoperative course was uncomplicated, and the patient was transferred to inpatient rehabilitation.

**Figure 1:**
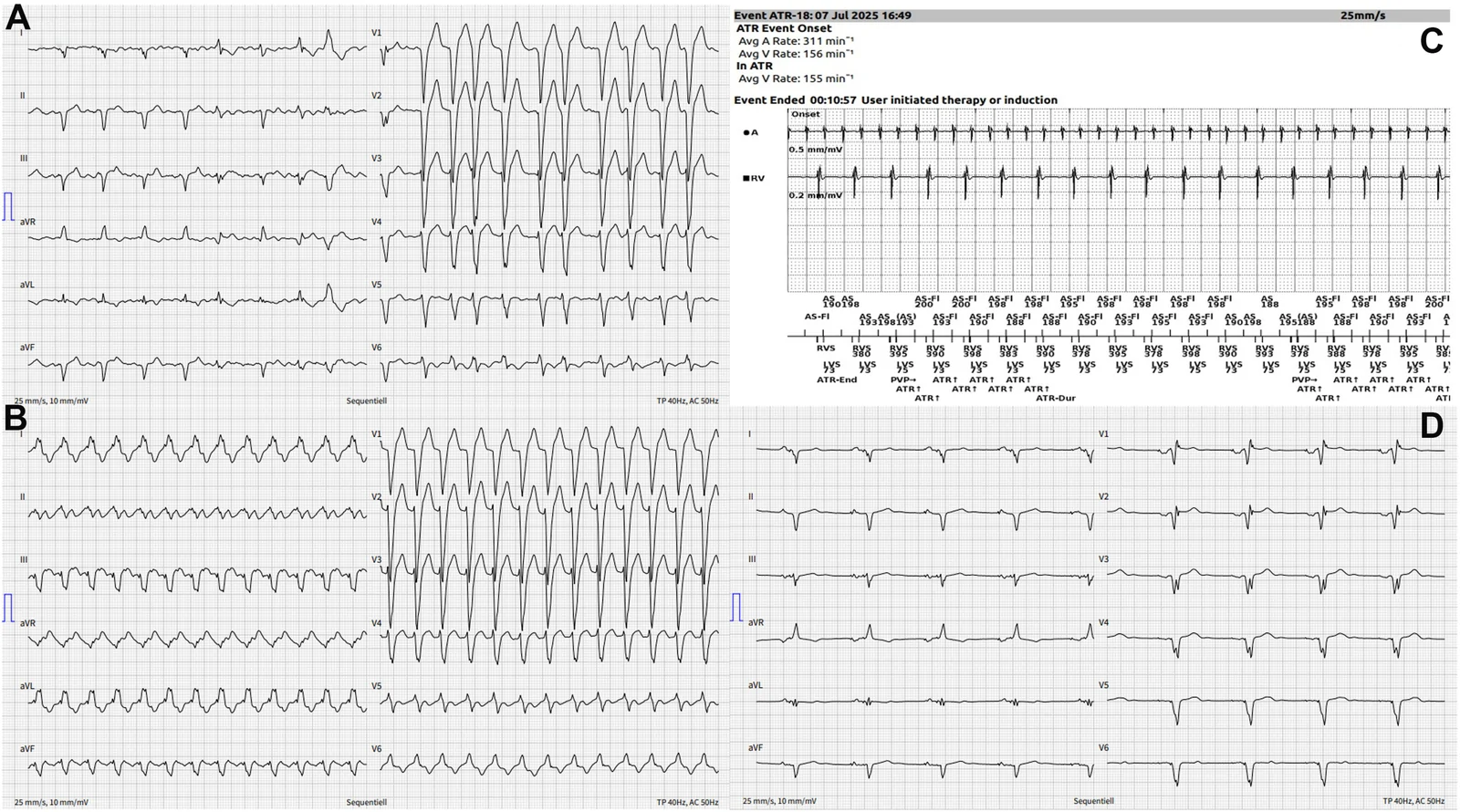
**A:** Electrocardiogram on admission showing atrial fibrillation. **B:** Electrocardiogram after conversion from atrial fibrillation to atrial tachycardia. **C:** Intracardiac electrogram of the atrial tachycardia from device interrogation. **D:** Sinus rhythm with biventricular pacing after conversion

On July 7, 2025, the patient was readmitted from the rehabilitation clinic to our tertiary care center with palpitations, thoracic discomfort, and New York Heart Association class III dyspnea. Electrocardiogram (ECG) on readmission revealed broad complex tachycardia compatible with atrial fibrillation (Figure 1A), which was confirmed with intracardiac electrograms in the subsequent device control. During the control, the tachycardia switched to an AT with 2:1 conduction (Figure 1B and 1C). Overdrive pacing failed to convert the tachycardia, but sinus rhythm was subsequently restored by electric cardioversion (Figure 1D). Amiodarone was started for rhythm control management. The patient had no history of atrial arrhythmias before the aortic valve reoperation.

**Figure 2:**
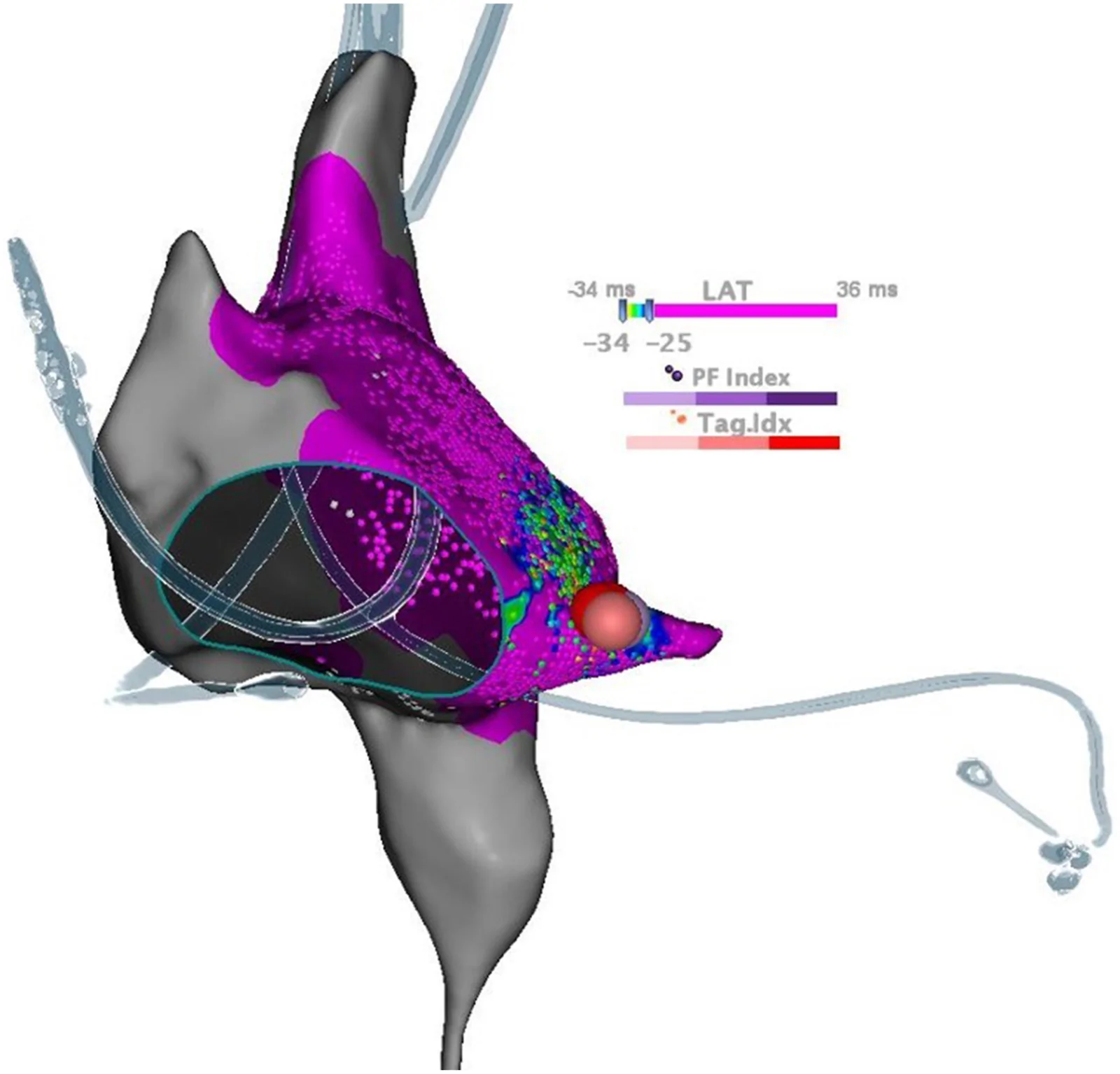
Activation map of the right atrium in LAO projection with CT overlap of implanted leads. Color gradient from *red* to *violet* indicates the earliest to the latest atrial electrogram sites. *Red ablation points* indicate radiofrequency lesions. *Violet ablation points* indicate PFA lesions at the CS ostium. CS = coronary sinus; CT = computed tomography; LAO = left anterior oblique; LAT = local activation time; PF = pulsed field; PFA = pulsed field ablation.

**Figure 3:**
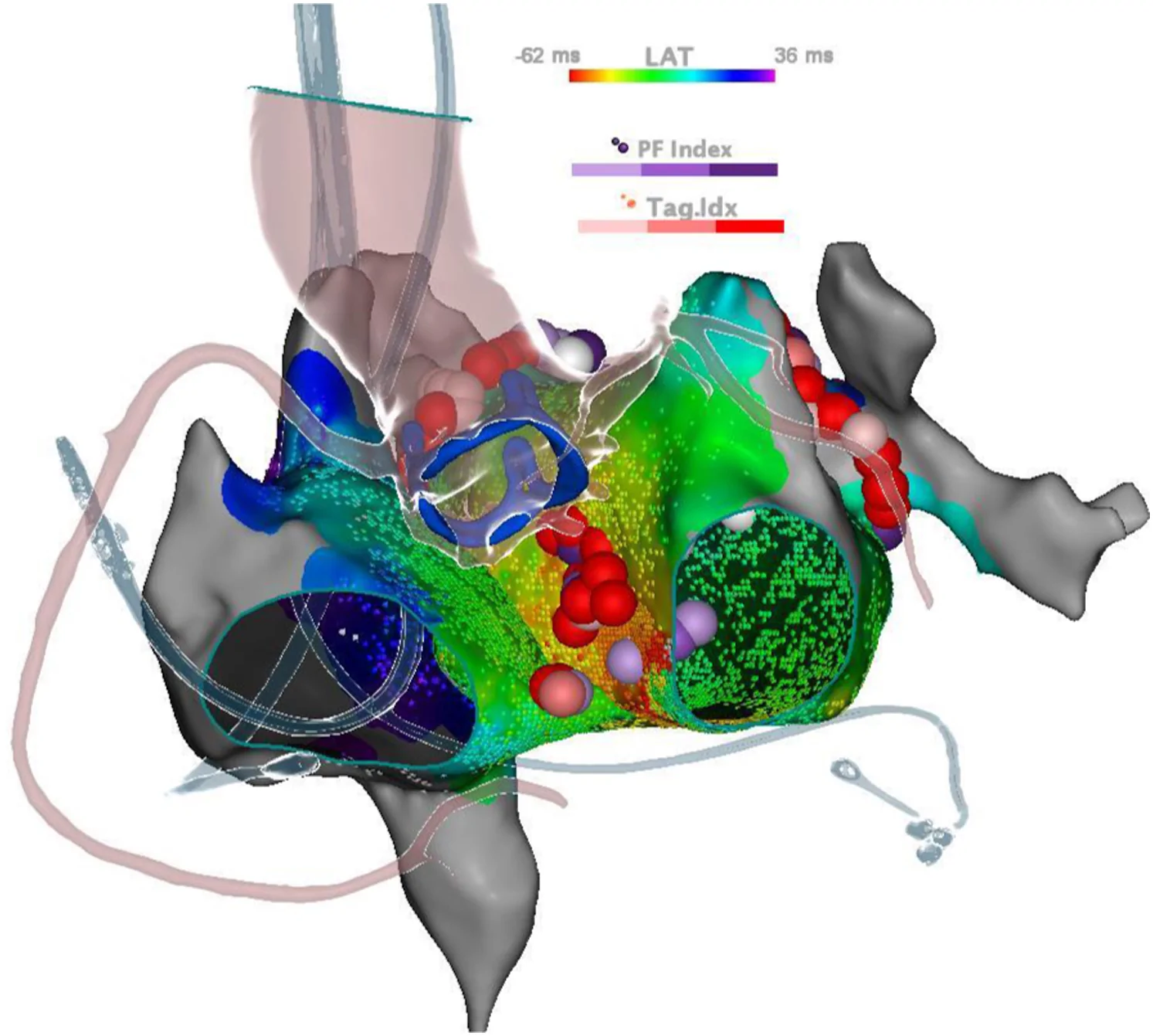
Activation map of the left atrium with CT overlap of implanted leads, ascending aorta with coronary arteries, and aortic valve prosthesis. Color gradient from *red* to *violet* indicates the earliest to the latest atrial electrogram sites. *Red ablation points* indicate radiofrequency lesions. *Violet ablation points* indicate PF ablation lesions. CT = computed tomography; LAT = local activation time; PF = pulsed field.

**Figure 4:**
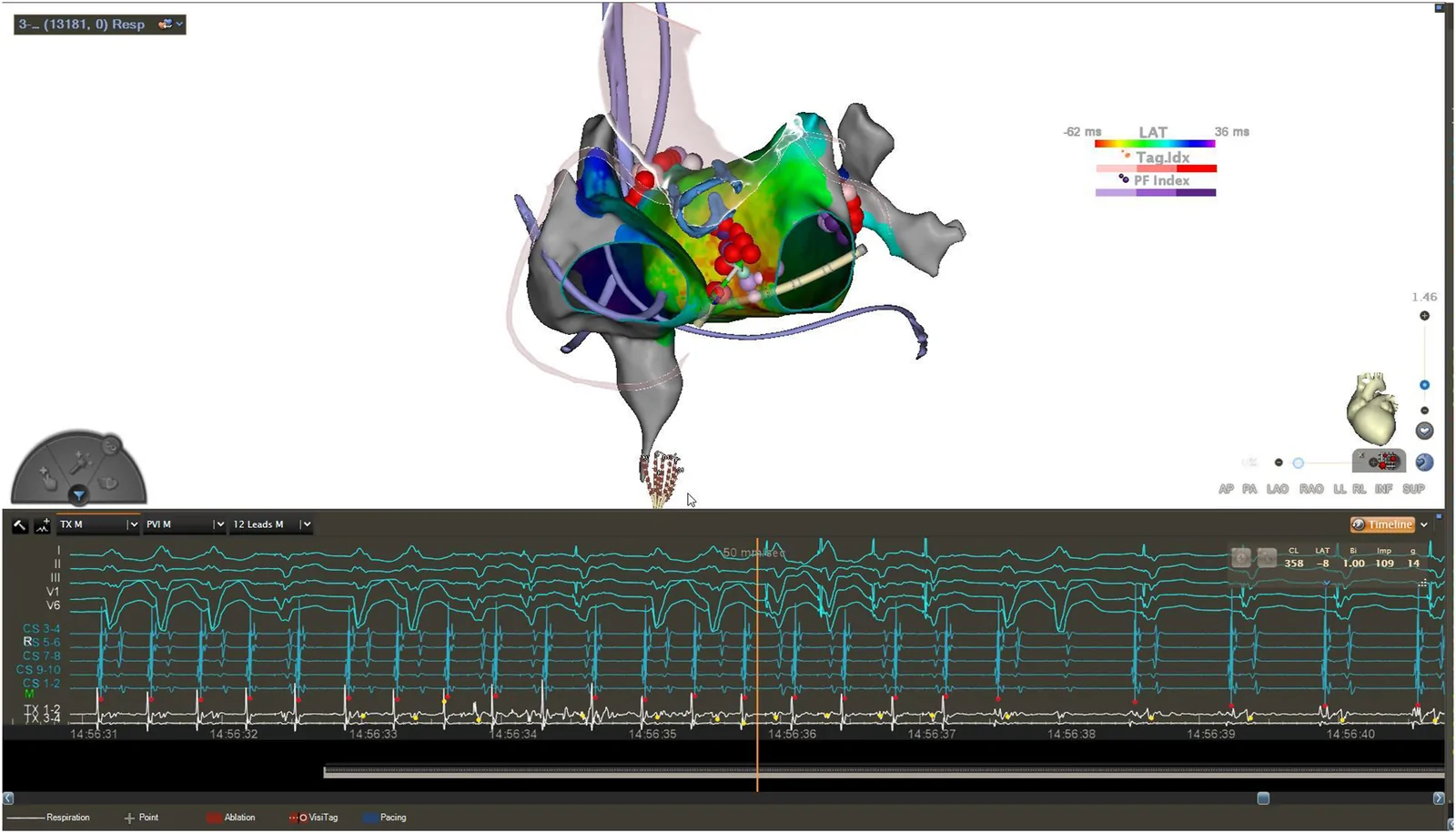
Termination of atrial tachycardia with radiofrequency application. *Top:* The activation map of both atria. *Bottom:* The surface electrocardiogram and intracardiac electrograms from the coronary sinus catheter indicating termination of the atrial tachycardia. LAT = local activation time; PF = pulsed field.

Owing to the recurrence of clinically relevant AT despite amiodarone therapy, the patient was scheduled for an electrophysiological study, pulmonary vein isolation (PVI), and catheter ablation of the AT. After transseptal access and a target activated clotting time of 350 seconds, electroanatomic mapping and 3-dimensional reconstruction of the left atrium (LA) were performed using an Octaray™ catheter (Biosense Webster, Irvine, CA). A retroaortic approach was not performed owing to concerns about the recent aortic valve replacement. The primary ablation strategy was PVI, and the secondary was AT ablation if the clinical AT was inducible. PVI was performed using radiofrequency (RF) ablation anterior (45 W; ablation index 550) and PFA (dual-energy THERMOCOOL SMARTTOUCH™ SF catheter, Johnson & Johnson, New Brunswick, NJ) posterior (900 V biphasic unipolar pulses; ablation index 400) according to the recommendation of the manufacturer. We then induced the clinical AT with changing cycle lengths. The earliest activation was mapped to the anterior LA and the anterior-superior CS ostium in the right atrium, consistent with a reentry circuit involving the aortic root. No entrainment maneuvers were performed owing to AT instability. Focal RF ablation and PFA at the anterior LA terminated the AT, but the arrhythmia remained reinducible. Therefore, we performed additional ablation with RF and PFA at the CS ostium in close proximity to the LV lead of the CRT system (distance to lead 9–10 mm), which terminated the AT with previous cycle length prolongation (with RF application) and eventually sustained noninducibility after consolidation with PFA. Ablation at the CS ostium was performed under close ECG monitoring to detect any potential changes in the paced ECG or repolarization abnormalities suggestive of a coronary complication. Activation maps and lesions are presented in Figures 2 and 3. Termination of the AT using RF is presented in Figure 4. Postinterventional device control revealed no abnormalities (Table 1). The patient was discharged in a stable condition, and no AT or atrial fibrillation recurrences were reported during the 6-month follow-up.

**Table 1:** Device control before and after ablation showing stable battery status and normal measurements for all implanted leads.

Parameter	Before ablation	After ablation	2 mo after ablation	6 mo after ablation
Battery status	10 y	9.5 y	∗	∗
RA lead				
Impedance RA	452 Ω	511 Ω	623 Ω	624 Ω
Sensing RA	3.7 mV	6.9 mV	8.3 mV	8.1 mV
Pace threshold RA	0.7 V / 0.4 ms	0.7 V / 0.4 ms	0.8 V / 0.4 ms	0.8 V / 0.4 ms
RV lead				
Impedance RV	637 Ω	628 Ω	687 Ω	670 Ω
Sensing RV	>25 mV	23.7 mV	25 mV	25 mV
Pace threshold RV	0.8 V / 0.4 ms	0.8 V / 0.4 ms	0.6 V / 0.4 ms	0.6 V / 0.4 ms
LV lead				
Impedance LV	716 Ω	380 Ω	496 Ω	533 Ω
Sensing LV	>25 mV	>25 mV	∗	∗
Pace threshold LV	0.7 V / 0.4 ms	0.8 V / 1.0 ms	0.5 V / 1.0 ms	0.5 V / 1.0 ms

LV = left ventricular; RA = right atrial; RV = right ventricular.

∗ Specific values for this parameter not available because the device control was performed by the treating cardiologist.

## Discussion

In the current case report, focal PFA in close proximity to the LV lead of a CRT system at the CS ostium was feasible, terminated the AT successfully, and did not lead to device malfunction. To the best of our knowledge, no previous report on the interaction of the focal PFA catheter used in our case and CIED exists.

Interactions between PFA and CIEDs have been reported across several commercially available systems. In an ex vivo study, PFA with the Farapulse system (Boston Scientific Corporation) did not result in CIED or lead damage (45 PFA applications <5 cm from lead tip and <15 cm from generator in a saline bath).^9^ In vivo data showed the feasibility and safety of the same PFA system in CIED patients, although transient inhibition of ventricular pacing has been reported.^10^^,^^11^ With the lattice-tip catheter of the Affera system (Medtronic, Minneapolis, MN), case reports have described the induction of ventricular fibrillation during both PFA delivery and RF application.^12^^,^^13^ Moreover, 1 report documented irreversible CIED generator damage characterized by the development of a high current drain that rapidly depleted the device battery and necessitated generator replacement using the same PFA system.^6^ Another report from the same group noted a temporary indication of battery depletion that subsequently resolved without intervention.^6^ Furthermore, ablation procedures performed with the Affera system, as well as with the Farapulse system, have been shown to produce electromagnetic interference with the potential of pacing inhibition, which may pose a particular risk to pacemaker-dependent patients.^8^ These emerging clinical reports suggest that interactions between PFA and CIEDs may vary across different PFA systems, potentially influenced by technical factors such as the use of monopolar vs bipolar energy delivery.

In our case, we opted for an ablation owing to recurrent, highly symptomatic AT unresponsive to amiodarone therapy. The involvement of the anterior LA and the anterior-superior CS ostium was consistent with a reentry circuit involving the aortic root. Owing to the recent redo aortic valve procedure, ablation in the aortic root was not possible. The patient was informed of the limited evidence supporting an approach of PFA in close proximity to implanted leads. Eventually, PFA using a dual-energy contact force–sensing catheter was used to perform ablation at the anterior LA and, because of insufficient suppression of the AT, also at the CS ostium, ultimately leading to termination and noninducibility of the arrhythmia using both RF and PFA. Notably, this was achieved despite the presence of a CRT system, without postinterventional evidence of lead or generator damage. Given the patient’s pacemaker dependency, we monitored him overnight on the cardiology ward and conducted another device control before discharge, which showed no abnormalities, except for a marked drop in LV lead impedance. Given the otherwise unremarkable device control without evidence of inappropriate sensing, no lead damage was suspected. A potential explanation would be an impedance drop owing to increased cell permeability after PFA, which may transiently alter tissue conductivity, although local impedance drops after PFA were reported to be significantly lower in the swine model.^14^ LV lead impedance recovered during follow-up, with all measurements remaining within the reference range at all times. A possible explanation for the absence of lead damage in this case may relate to the specific electrical characteristics of the dual-energy catheter. Compared with other PFA systems, this catheter operates at a lower voltage and delivers focal, unipolar pulses. As a result, the highest electric field intensity is concentrated at the 3.5 mm tip, with a rapid decay in field strength as the distance from the tip increases. This spatial distribution may limit the exposure of adjacent structures, such as the LV lead, to clinically significant electric fields even when ablation is performed in relatively close proximity. Supporting this concept, previous observations in PFA have demonstrated that certain complications, such as coronary vasospasm, are primarily dependent on local electric field intensity rather than pulse number, underscoring the importance of field strength distribution over total energy delivery.^15^ Nevertheless, dedicated preclinical studies are required to further validate this hypothesis.

## Conclusion

This case demonstrates the safe and effective use of focal PFA using a dual-energy catheter at the CS ostium in a patient with an LV lead. Despite the anatomic challenges, arrhythmia termination was achieved without device dysfunction. Interaction between PFA and CIEDs may vary across different PFA systems with polarity of the current delivery potentially playing a crucial role in determining the nature and extent of device interaction. No general conclusions regarding safety can be drawn, and further systematic evaluation is required.

## Disclosures

Patrick Badertscher has received research funding from the “University of Basel,” the “Stiftung für Herzschrittmacher und Elektrophysiologie,” the “Freiwillige Akademische Gesellschaft Basel,” the “Swiss Heart Foundation,” and Johnson & Johnson and reports personal fees from Bristol Myers Squibb (BMS), Boston Scientific, and Abbott, all outside the submitted work. Michael Kuehne reports grants from the Swiss National Science Foundation (grant numbers 33CS30_148474, 33CS30_177520, 32473B_176178, and 32003B_197524), the Swiss Heart Foundation, the Foundation for Cardiovascular Research Basel, the University of Basel, Bayer, Pfizer, Boston Scientific, BMS, and Biotronik and grants and personal fees from Daiichi Sankyo, all outside the submitted work. Christian Sticherling is a member of Medtronic Advisory Board Europe and Boston Scientific Advisory Board Europe and received educational grants from Biosense Webster and Biotronik and a research grant from the European Union’s FP7 program and Biosense Webster and lecture and consulting fees from Abbott, Medtronic, Biosense Webster, Boston Scientific, MicroPort, and Biotronik, all outside the submitted work. Philipp Krisai reports speaker fees from BMS/Pfizer and grants from the Swiss National Science Foundation, Swiss Heart Foundation, Foundation for Cardiovascular Research Basel, and Machaon Foundation. The other authors have no conflicts of interest to disclose.
